# Supplementary material for: Assessing parent-child interaction with deaf and hard of hearing infants aged 0–3 years: An international multi-professional e-Delphi
Source: PLoS One. 2024 Apr 29;19(4):e0301722. doi: 10.1371/journal.pone.0301722 (PMC11057743; doi:10.1371/journal.pone.0301722)
Supplement: S1 Table — (DOCX) [file pone.0301722.s001.docx]

**S1 Table: Reworded statements from Round 1 used in Round 2**

For uniformity, statements have been edited into the third person, i.e. ‘Parent using visual attention-getting strategies’ to ‘Parent uses visual….’

| **Parent Behaviours: Round 1 Statements** | **Reworded statements used in Round 2** |
| --- | --- |
|  | **These behaviours had the following statement before them:*  This parent behaviour may be particularly relevant for children who are visually motivated and/or benefit from access to lip patterns / facial expressions. The use of these parent behaviours will be influenced by the child’s hearing status, their access to spoken language (over distance/noise), use of audiological devices, and/or the chosen language(s) used by the family. |
| PB1 Parent uses visual attention-getting strategies (i.e., waving). | *PB1: Parent uses visual attention-getting strategies (e.g., moving into the child’s visual field). |
| PB3 Parent uses tactile attention-getting strategies (i.e., tapping the floor). | *PB3: Parent uses tactile attention-getting strategies (e.g., tapping). |
| PB4 Parent uses multi-modal attention-getting strategies (i.e., tapping the child and saying 'look'). | *PB4: Parent uses multiple strategies at one time to gain the child’s attention (e.g., moves into the child’s visual field and says ‘wow’, taps and says the child’s name). |
| PB5 Parent actively waits or pauses their communication until their child looks at them. | *PB5: Where the child benefits from access to lip patterns, facial expressions, and/or visual perception of sound, parent actively waits or pauses their communication until their child looks at them. |
|  | *+ These behaviours had the following statement before them:* This parent behaviour may be particularly relevant for children who have access to sound and the potential to understand and use spoken language. |
| PB2 Parent uses auditory attention-getting strategies (i.e., saying 'wow') | +PB2: Parent uses auditory attention-getting strategies (e.g., using the child’s name). |
| PB21 Parent uses appropriate voice volume. | +PB21: Where the child is using/developing skills in spoken language, parent uses appropriate voice volume. |
| PB22 Parent is mostly within 1 to 2 meters of amplification device(s). | +PB22 Where the child is using/developing spoken language, parent is mostly within 1 to 2 meters of the child’s amplification device(s) where possible. |
| PB23 Parent makes accompanying sounds to the child’s action / toys / items. | +PB23 In earlier stages of development, where the deaf child has access to spoken language, parent makes accompanying sounds to the child’s actions / toys / items. |
|  | *The below final statements were introduced with the following sentence:*  The following statements are to be considered no matter the family’s choice in spoken and/or signed languages. |
| PB7 Parent stays still or silent when the child looks away. | PB7 Parent watches and waits when the child looks away, to allow the child to explore, to take a rest from interacting, to allow the child to take a turn in initiating. |
| PB12 Parent spends time observing their child's behaviours and initiations, before using language. | PB12 Within the interaction, parent waits and watches their child’s behaviours and gaze, using the child’s cues to tailor the language they will use. |
| PB28 Parent uses mental state verbs (i.e., 'like', 'know', 'think') within the interaction. | PB 28 Where contextually and pragmatically appropriate (developmental stage / relevant moment), parent uses mental state verbs (i.e., 'like', 'know', 'think') within the interaction. |
| PB29 Parent describes actions or events ahead of doing them. | PB29 Parent informs the child of an action or event ahead of doing it, using a range of visual cues if appropriate for the child’s understanding. |
| PB30 Parent reduces questions and increases on-topic comments. | PB30 Parent uses open questions in favour of closed questions. |
| PB32 Parent rephrases the child's language with correct grammar. | PB32 Within the interaction, parent supportively rephrases the deaf child’s language with correct grammar (where contextually and pragmatically appropriate, i.e., developmental stage, a natural moment). |
| PB33 Parent rephrases their child’s language into a question. | PB33 Parent rephrases their child’s language into a question, i.e., the child says/signs "cake" and the parent rephrases into "Can I have cake, daddy?’ |
| PB34 Parent uses open questions. | PB34 Parent balances open questioning with on-topic comments. |
| PB37 Parent models mistakes in their own language use. | PB37 Parent models mistakes in their own language if/when they arise, i.e., ‘The fireman is crying... I mean climbing! I used the wrong word/sign’. |
| PB39 Parents’ average number of signs / words used in interactions to be noted. | PB39 Parent provides language input (i.e., average number of signs/ words) that is appropriate to child’s developmental stage. |
| PB40 Parent's frequency and use of touch. | PB40 Parent uses touch as a tactile way of highlighting speech / tone / rhythm in their language (e.g., parent says ‘Hel-lo Ma-ya’ with taps for each syllable). |
| **Approaches to Assessment: Round 1 Statements** | **How They Were Reworded in Round 2** |
| AA41 Joint engagement should be evaluated by number of interactive turns between parent and child. | AA41 Evaluating joint engagement could be observing the connected turns between parent and child. In some cases, it may be appropriate to count these turns. |
| AA42 Joint engagement should be measured by total length of time engaged. | AA42: Joint engagement could be observed by noting how long a parent and child remain connected. In some cases, it may be appropriate to estimate this, particularly for the purpose of reviewing progress. |
| AA43 The parent-child interaction assessment should be recorded on video. | AA43 To accurately capture and then reflect on parent-child interaction, a video recording is recommended at least once in parent/professional partnership work. Timing of when this formal measure is taken will depend on parental well-being, parental personality and the strength and trust within the parent/professional relationship. |
| AA44 Video recordings should be less than 10 minutes in length. | AA44 Though a video recording of 10 minutes of interaction should provide enough material for watch back and reflection, the length of a video recording should be discussed with parents as they may request more or less time. |
| AA45 Parents should be encouraged to send videos to an early intervention provider for review of more natural interaction, where the professional is not present (especially if the child has additional needs). | AA45 Parents could be encouraged to send videos to an early intervention provider for review, where the professional is not present (especially if the child has additional needs, the child does not engage, or parents require support within a particular context). |
| AA47 Assessments should take place in the child and parents’ most natural setting. | AA47 Where possible, assessments of parent-child interaction should take place in the child and parents’ most natural, most familiar settings. |
| AA48 Parents should be asked where they would prefer to be assessed. | AA48: Where possible, parents should be asked where they would prefer to be observed. |
| AA49 As well as play, parent-child interaction should be assessed within daily routines (i.e., dressing, mealtimes). | AA49: As well as observing interaction in play, professionals could sample interactions within daily routines (e.g., mealtimes, dressing) where parents are willing. |
| AA50 For children with additional or complex medical needs, assessments may need to focus more on everyday routines and care, compared to play. | AA50 For some children, e.g., those with additional or complex medical needs, the activities within parent-child interaction assessments may need to be more flexible and varied, i.e., whenever the child is most interactive within their daily routines. |
| AA52 The entire review of the assessment should be strength-based. | AA52: The review of the parent-child interaction assessment should be largely strength-based, i.e., identifying what is working well. There could also be scope to sensitively highlight behaviours with potential to improve, as long as the overall review is positive and encouraging. |
| AA54 Scales should be used to observe each parent behaviour (i.e., not observed, emerging, present). | AA54: Parents and professionals could reflect on each parent behaviour together using scales. Professionals could describe each parent behaviour before the parent reflects on their interactions. The wording of the scale to be parent-centred and positively framed. |
| AA56 Parents should be sent a copy of the assessment video, following the assessment session with the professional. | AA56: Where possible, the parent should be offered the choice of receiving a copy of the parent-child interaction recording, following the assessment session with the professional. |
| AA57 All main caregivers (i.e., mothers, fathers, grandparents) should be given the opportunity to have their interaction skills reviewed. | AA57: If the family requests or the context deems it necessary, all main caregivers (i.e., mothers, fathers, grandparents, older siblings) should be given the opportunity to have their interaction skills observed and reflected upon. |
| AA60 Professionals (i.e., Early Interventionists, Family Support Workers, Teachers of the Deaf and / or Speech and Language Therapists) should conduct joint assessments of parent-child interaction where possible. | AA60: Where a family has more than one professional involved, the assessing professional should share information from the assessment with the rest of the team. This will reduce duplication of assessment and allow quicker access to intervention / support. |
| AA61 Families who do not share the same language as the assessor require bilingual co-workers or interpreters for the assessment, but the parent behaviours being assessed do not require adaptations. | AA61a Families should have their parent-child interaction observed in the language of the home, with assessors using interpreters or bilingual co-workers to understand the language used. |
|  | AA61b The culture of the family should be acknowledged when observing parent-child interaction, to prevent the professional misinterpreting assessment results. |
|  | AA61c All parents, even where there may be cultural differences at play, may benefit from adapting their communication behaviours if unhelpful for the language development of the deaf child. The review of an assessment video can assist with these discussions. |
| AA64 Goals should be focused on a parent’s current strengths in the assessment. | AA64: Goals should be mostly focused on a parent’s current strengths in the assessment. The parent may also wish to pick an important behaviour they would like to practice / become more confident with. |
