# Supplementary material for: Assessing parent-child interaction with deaf and hard of hearing infants aged 0–3 years: An international multi-professional e-Delphi
Source: PLoS One. 2024 Apr 29;19(4):e0301722. doi: 10.1371/journal.pone.0301722 (PMC11057743; doi:10.1371/journal.pone.0301722)
Supplement: S2 Table — (DOCX) [file pone.0301722.s002.docx]

**S2 Table: Number of statements per category achieving consensus across the E-Delphi study**

The tables below show how many statements from each category achieved consensus in rounds 1 and rounds 2. Following round 2, a further six skills were included following data analysis and review.

Parent Behaviour Categories

| **Category of Parent Behaviour (number of PB statements)** | **Achieved Consensus in Round 1** | **Achieved Consensus in Round 2** | **From final review**  **discussion** | **Included Statements** | **No Consensus (i.e., discarded)** |
| --- | --- | --- | --- | --- | --- |
| Attention Getting (n=5) | 0 | 3 | 0 | 3/5 | 2 |
| Joint Engagement (n=4) | 4 | N/A | N/A | 4/4 | - |
| Parental Sensitivity (n=8) | 8 | N/A | N/A | 8/8 | - |
| Access to Language (n=4) | 1 | 2 +1 added | 0 | 4/5 | 1 |
| Language Enrichment (n=19) | 8 | 7 | 2 | 17/19 | 2 |
| **Total** | **21** | **13** | **2** | **36/41 (88%)** | **5/41**  **(12%)** |

Approaches to Assessment Categories

| **Category (number of AA statements)** | **Achieved Consensus in Round 1** | **Achieved Consensus in Round 2** | **From final review**  **discussion** | **Included Statements** | **No Consensus**  **(discarded)** |
| --- | --- | --- | --- | --- | --- |
| Assessment Set Up (n=3) | 0 | 0 | 2 | 2/3 | 1 |
| Measuring Skills (n=4) | 0 | 0 | 0 | 0/4 | 4 |
| An Informed Parent (n=4) | 2 | 2 | N/A | 4/4 |  |
| An Empowered Parent (n=4) | 1 | 3 | N/A | 4/4 |  |
| Collaborating with Parents (n=3) | 0 | 1 | 2 | 3/3 |  |
| Goal setting (n=4) | 4 | N/A | N/A | 4/4 |  |
| Multi-Professional Joint Working (n=2) | 0 | 1 | N/A | 1/2 | 1 |
| Cultural Diversity (n=1) | 0 | 1+2 added | N/A | 3/3 |  |
| Deaf-Plus (n=1) | 0 | 1 | N/A | 1/1 |  |
| **Total** | **7** | **11** | **4** | **22/28 (79%)** | **6/28**  **(21%)** |
