# Supplementary material for: Assessing parent-child interaction with deaf and hard of hearing infants aged 0–3 years: An international multi-professional e-Delphi
Source: PLoS One. 2024 Apr 29;19(4):e0301722. doi: 10.1371/journal.pone.0301722 (PMC11057743; doi:10.1371/journal.pone.0301722)
Supplement: S1 File — (DOCX) [file pone.0301722.s003.docx]

**S1 Participants’ Feedback for Round 2 Open Text Question: When observing PCI with deaf children with additional needs, are there any methods that you have found useful?**

All non-identifiable contributions have been added here, grouped into similar points.

**Consulting with parents:**

- Using parents to make and send their own videos through to the professional
- Listening to parent/carer about what the child particularly likes or engages with.
- Spending time getting to know the family first. Finding out background information maybe through an interpreter so you know the child's routine and language community.
- Professional taking carer advice on time of day, duration, activity, and location where most interaction happens.
- Check when the best time to do the session would be. For these sessions I would say home visits can work better.
- Important to work from where the parents are. Seek their advice on which routines or activities they believe will work best rather than setting the activity. Incredibly important to address it using the key principles of appropriate support.
- Really knowing the child and asking parents for their thoughts
- Making sure to seek parental insights and input about what works, what doesn't work -- even if the professional doesn't agree, it's good to respect the parents as the "experts" about their own child.
- Asking the parent where they feel communication is strongest and where they could use more support in determining best ways to communicate successfully and then observing both situations
- You've touched on it in how you've worded AA50 - work with parents to discern what time of day/what type of interaction is best for them and their child.
- Negotiating with parents the best time of day. Joint visits with other professionals as they often have so many involved.
- Find out when the child is happy and record that.
- No one method works for all. I have found it's best to work co-operatively and collaboratively with parents, trying a range of approaches that fit best into their daily lives and take account of their child's needs. In my view the 'best' approach is the one that the family feels the most comfortable with and that works best to yield the maximum information to inform support. EI practitioners need to try any and all approaches to find, with the family, what works best for them. Diaries, videos (especially when the child is enjoying an activity) and at different times of day can all work

**General adaptations (or none):**

- Shorter interactions
- Observing during daily routines
- Observe their natural interactions
- No, PCIT principles are the same regardless
- Child in their home environment
- Observe in group sessions e.g., during a coffee morning when child is relaxed.
- I have used a number of systems - I have found making a video at home and sending in for joint review over MS teams has been a positive move forward
- If processing time and eye contact is not offered then other methods of PCI will need to be established as 'normal behaviour'. This may need to be observed over a much longer period of time and may not be evident or captured in 10 minutes.
- Encouraging the carers to do what the child likes best e.g., people games, intensive interaction, sensory play etc
- Being flexible is key, observing PCI in different scenarios such as nursery, hospital, home and even outdoors.
- Finding activities that the parent and child truly both enjoy. Get away from 'setting up a learning situation' - just both have fun -emphasis on enjoying each other's company.
- Using online sessions has proved to be beneficial as the parent can have their phone set up anywhere in the house where they would prefer the observation to take place.
- Observing over a longer time frame (within session as well as on more frequent visits) and taking care to acknowledge more subtle responses. A variety of settings. Agree that daily routines are key here.
- Although it isn't popular now, we found lots of good info with dyadic toy play. Home based activities like preparing for dinner, hygiene, also good. Need some observation with objects, others without object focus.
- Looking at anticipating each other within familiar routines e.g., both how the parent interprets the child's subtle signals such as change in breathing, muscle tone - and also how the child anticipates/responds to next steps within familiar routines e.g., tilting chin up towards spoon during feeding

**Physical factors to consider:**

- Encouraging the parent to consider positioning of the child i.e., on the floor or in adapted seating/standing frame etc.
- Think about child's mobility - ensure they are comfortable
- Having the child sat in a chair with a tray for toys/books to be placed on helps to contain the child while interacting if the child finds it hard to sit still for less than a few minutes.
- Parent at same level, up close and personal
- Consider any support resources e.g., seating, splints, light or sound management that may be needed. Depending on additional need, interaction with a range of carers may be helpful

**Resources and Assessments:**

- Triple-C-Checklist; observing the milestones of prelinguistic interaction like (pre-)intentionality and (pre-)symbolic play; additionally try out of methods of augmented and alternative communication
- Video interaction guidance and It Takes Two to Talk (HANEN)
- I have used the Affective Communication assessment and the Behavioral Observation of Hearing, to look at very complex children and to ascertain if we are getting responses to sound or interaction
- Approaches by Jan van Dijk (not only for deafblind children)
- The ES Developmental Journal done with the parents only using the relevant section/pages.
- Observations by other professionals
- Having an accompanying person/professional to observe session
