# Supplementary material for: Assessing parent-child interaction with deaf and hard of hearing infants aged 0–3 years: An international multi-professional e-Delphi
Source: PLoS One. 2024 Apr 29;19(4):e0301722. doi: 10.1371/journal.pone.0301722 (PMC11057743; doi:10.1371/journal.pone.0301722)
Supplement: S2 File — (DOCX) [file pone.0301722.s004.docx]

**S2 Participants’ Feedback for Round 2 Open Text Question: Please describe how to successfully manage assessments with families who use their home language to communicate with their deaf child (a language different from that of the country they reside in).**

All non-identifiable contributions have been added here, grouped into similar points.

**Interpretation / Translation**

- In children 0-3 a monolingual professional can observe a lot, even, if he/she does not speak the language, especially the preverbal aspects of communication development. This can serve as a base. If translation is additionally available, this would be better, but not essential. In addition questionnaires in the families language can be helpful!
- As with many of these questions, it is important to keep sessions as straightforward and uncomplicated as possible in order to be realistic regarding how they will work in a range of work settings. If an interpreter is already present for parents, it makes sense to ask them about the language used. However, caution should be taken that an interpreter communicates exactly what has been said/type of language and vocabulary used so time needed to explain this. If parents also speak English, may not feel appropriate to book an interpreter. If there is a bilingual colleague available, that's great but might not be general practice depending if a variety of languages are used among parents. Some behaviours, e.g., length of connected turn, way parents gain attention can still be observed in another language
- By getting the free play recording translated
- I think the interpreter needs careful briefing and if possible be attached to the team so they understand the whole aim of doing observations about language and interaction in the home. I have done this once or twice with spoken language interpreters with deaf / deafblind children aged 0 - 5. I think it would be better to have the bilingual professional lead - but an interpreter is not an early years language worker. Training time would need to be built in. They may have preconceived ideas about language development just as parents do too and other support staff.
- Including researchers/professionals fluent in the language observed.
- I have only been able to use interpreters and we have carried assessments out together with a lot of guidance. It is very challenging and time consuming to get it right.
- We use bi-lingual therapists but are lucky to have them in our center; they speak English and Spanish
- I have delivered a 3 month video intervention through an interpreter (spoken language) the outcomes were very successful for the family
- Have experienced difficulties when I've had to use a foreign language interpreter when an ESL teacher has not been available, it did not work as well, the teacher understood the purpose in a way the interpreter did not and they became too involved in the assessment.
- We have done meetings/sessions with an interpreter taking the lead who has been prepared by the professional regarding expectations of the interaction. The interpreter has then used their skills to help the monolingual professional.
- If the parent is confident in a language that the assessor is using then the parent can report back on what was being said. The actual content of the language used is only a fraction of PCI
- Interpreter in the session F2F or joining remotely
- Discuss with parents with help from the interpreter present - they would have to be part of the reflection of what had been noted in the video
- ’Supporting deaf children who speak English’ as an additional language document. Look at section on use of interpreters.
- I often watch it back with the parent and get them to tell me what they have said if their English is good enough. If not, I have had the interpreter tell me what was said. It takes longer of course.
- I find this challenging. In my experience, you need an appropriately trained co-worker to facilitate this - it doesn't always work well with interpreters.
- Working alongside a sign communicator to assess the video after the event
- Involving family members, from time to time online professional interpretation works, helps a lot when it is possible to have some time for explanations with the interpreter and when the same interpreter is used again and again
- I’ve used student SLTs who speak the family language to join me on appointments.
- Sharing the lead depending on the language levels of the child and parent. If child pre-linguistic, bilingual co-worker explains activities to parent as co-worker completes non-linguistic tasks - a mixture of these approaches
- We have used interpreters. this can be incredibly successful, but we also have had situations which have been difficult due to the level of understanding of the work by the interpreter, and also the familiarity of the interpreter. Where the family have a choice and can request a specific interpreter they feel comfortable with then this has also worked well. This has built up professional understanding and mutual respect between all parties which is so important
- Provide bilingual assessors as a first preference, or interpreters as a second tier option
- Training/discussion on PCIT with interpreter so they are aware of goals and aims & translate accordingly
- Sometimes the parents can speak English. Otherwise we also tried digital translator tools. Or we invite other families with the same language who can help to translate. Our experience is that professionals are very good at assessing language behaviour, whether they understand the language or not. It is only necessary for the subsequent counselling.

**Other**

- Encourage home language interactions through songs and home books
- Interaction is generally language and cultural independent. I will only ask if a certain action is considered appropriate in their language and culture.
